# Study on the Nodal Composite Bearing Performance of Nontruncated PHC Pipe Pile and Bearing Platform

**DOI:** 10.3390/ma17133216

**Published:** 2024-07-01

**Authors:** Yasheng Liu, Zhaosheng Guo, Wubin He, Xinsheng Ge, Jingyuan Sun

**Affiliations:** College of Civil Engineering, Taiyuan University of Technology, Taiyuan 030024, China; lyscivil@163.com (Y.L.); hewubin@tyut.edu.cn (W.H.); gxstyut@126.com (X.G.); sunjingyuan0074@link.tyut.edu (J.S.)

**Keywords:** nontruncated pile, pipe-pile cap connection, reciprocating load test, bearing capacity, numerical simulation, embedded depth

## Abstract

In this paper, low circumferential reciprocating load foot-scale tests were performed on two nontruncated PHC B 600 130 tubular piles with bearing nodes to characterize the damage process and morphology of the specimens and to investigate the load-carrying performance of the members. The test results reveal that under the action of tensile-bending-shear loading, the bearing concrete in the node area buckles and is damaged, the anchored reinforcement in the node area yields, the constraint is weakened, an articulation point is formed, and the node rotational capacity increases. When the embedment depth increases from 200 mm to 300 mm, the ultimate bearing capacities of the positive and negative nodes increase by 31.04% and 36.16%, respectively. A numerical simulation is used to verify the test results. Considering the four types of piles without truncated nodes, the numerical simulation is used to analyze the node-bearing capacity at different embedment depths. Finally, a preferred node type is proposed as follows: a terminal plate welded anchor bar and pipe pile core-filled longitudinal reinforcement anchored into the bearing node, with a preferred embedment depth of 250 mm.

## 1. Introduction

Compared to cast-in-place piles, cement fly-ash gravel (CFG) piles, cement mixing piles, and other foundation types, pre-stressed high-strength concrete (PHC) pipe piles have favorable compressive, flexural, and crack resistance; strong adaptability to geological conditions; reliable quality; favorable durability; favorable economic performance; fast construction; no mud pollution; no need for large area excavation; and other advantages. These elements have been widely used in existing construction projects. When PHC pipe piles are friction piles, the depth of pile sinking is often used as a control index representing engineering quality, and the pile sinking depth is controllable. Under these conditions, a PHC pipe pile at the connection with the cap does not need to be cut off. Because the node between the pile and cap is the concentrated part of the load transfer from the superstructure to the pile foundation, it is easily damaged under the action of earthquakes; in some cases, this damage is very serious [1,2,3,4,5]. Efforts have thus been carried out to study the bearing capacity of nodes between stakes and stake caps.

Xu Y. et al. [6] reviewed the current research status of a socketed column and bearing platform connection in bridge engineering, analyzed the influence of key design parameters of socketed connection structure on the mechanical behavior of the socketed structure, and discussed the methods to improve the seismic performance of the socketed structure. Takuya, N. et al. [7] reported brittle failure at the pile head during the 1995 Hyougoken-Nanbu earthquake. Roeder, C.W. et al. [8] studied three methods to improve the performance of octagonal precast concrete pile-wharf connections and proposed the controlled rotation connection. Zhao Cheng et al. [9] investigated the socketed connection members of non-prestressed reinforced concrete precast columns and bearing platforms, and the test results showed that the column connection and pile connections exhibited excellent behavior. Richards, P.W. et al. [10] investigated four full-scale specimens in the field under cyclic loading. The moment-bearing capacity of the pile–cap connection in the test is analyzed.

Lenci, K. et al. [11,12] and Zhang, G. et al. [13] proposed the development of an analysis methodology for predicting the ultimate seismic capacity of a concrete-filled steel tube for reinforced concrete pile cap connections.

Takuya, N. et al. [7]; Park, J.B. et al. [14]; Bang, J.W. et al. [15]; He, W.B. et al. [16]; and Yang, Z.J. et al. [17,18] studied the bearing performance of PHC pipe–cap connections before and after improvement through full-scale tests.

Guo, Z.S. et al. [19,20] studied the load-carrying capacity of PHC pile–platform connections through the utilization of full-size specimens and ABAQUS software and presented an equation for calculating the bearing capacity of pile–cap connections under horizontal earthquake action.

Previous efforts have more intensively studied the combination of bending–shear and compression–bending–shear forces, with less focus having been given to the combination of tension–bending–shear forces. However, the load characteristics of the transmission line foundation are complex, and these characteristics are subjected to the influences of a tension/pressure alternating load as well as a large horizontal load. The stability of anti-pulling and anti-overturning effects is usually the design control condition of a transmission line foundation. Therefore, based on the “Research on Key Technologies of pull-out and shear bearing performance of prestressed high-strength concrete pipe piles for Transmission Lines” project of the State Grid, this study investigates the tensile-flexural shear-bearing performance of PHC pipe piles connected to caps via full-scale tests and numerical simulations.

## 2. Experimental Design

**Figure 1:**
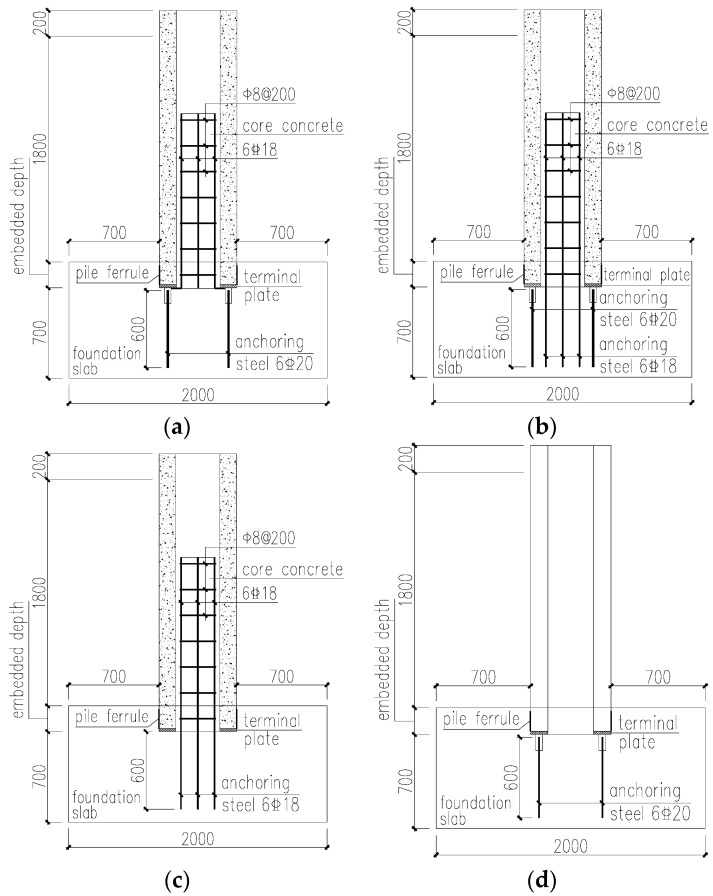
Four types of node diagrams. (**a**) The terminal plate welded anchor bar + core-filled longitudinal bar unanchored into the cap node (Type I node); (**b**) the terminal plate welded anchor bar + core-filled longitudinal bar anchored into the cap node (Type II node); (**c**) the core-filled longitudinal bar anchored into the cap node (Type III node); (**d**) the terminal plate welded anchor bar node (Type IV node).

For the connection mode between the PHC pipe pile and cap without cutting the pile, the current “Prestressed Concrete Pipe pile” [21] recommends that the terminal plate welded anchor bar + core-filled longitudinal bar are unanchored into the cap node. This node later becomes a Type I node, which is a common node type in engineering. With reference to Type I nodes, the present author designed the terminal plate welded anchor bar + core-filled longitudinal bar anchored into the cap node (Type II node), the core-filled longitudinal bar anchored into the cap node (Type III node), and the terminal plate welded anchor bar node (Type IV node). The detailed structure is shown in Figure 1. Compared with the Type I node, the Type II node anchors the filling longitudinal bar into the cap. Compared with Type I nodes, Type III nodes remove the terminal plate welded anchorage bar and anchor the filling longitudinal bar into the cap. To explore the node-bearing capacity of noncore-filled pipe piles, Type IV nodes are designed. Compared with Type I nodes, the core-filled concrete and rebar are removed from Type IV nodes.

### 2.1. Experimental Design

The authors designed two full-scale test objects of Type I nodes. In this paper, the stress capacity of non-cut-off PHC B 600-130-type PHC stakes [21] with bearing cap connecting nodes was investigated. The outer diameter of the pipe stake is 300 mm and the inner diameter is 170 mm. Table 1 shows the major dates of the test objects. The connecting plate welded to the terminal plate is made of Q235B steel, with a height of 120 mm, a width of 50 mm, and a thickness of 16 mm.

Six specimen blocks of each batch of concrete used for the tests were carried out for compressive strength tests. The results showed that the average compressive strength of the stake body concrete was 93.20 MPa, and the average compressive strength of the bearing cap and core filling concrete in CT-200 and CT-300 was 38.71 MPa and 40.30 MPa.

The rebar parameters in the specimen are listed in Table 2.

### 2.2. Fabrication of Specimens

PHC stakes were produced by Jianhua Building Materials (Shanxi) Co., Ltd. (Lvliang, China) and the cap and core filling were made by the Laboratory of the Research Institute of Bridge and Tunnel Engineering of Shanxi Transportation Science and Technology Research and Development Co., Ltd. (Taiyuan, China). For the CT-200 specimen, the length of the stake was 2.2 m. For the CT-300 specimen, the length of the stake was 2.3 m. The length of the test bearing platform was 2.0 m, width 1.2 m, height 0.9 m (CT-200) and 1.0 m (CT-300). The pipe stake was made of a B-type stake with a diameter of 600 mm, and the bearing table was equipped with HRB335 ϕ14@150/160 mm double-layer bidirectional reinforcement. During the test, the axial tension applied to the top of the stake was 340 kN.

### 2.3. Test Loading and Measuring Devices

**Figure 2:**
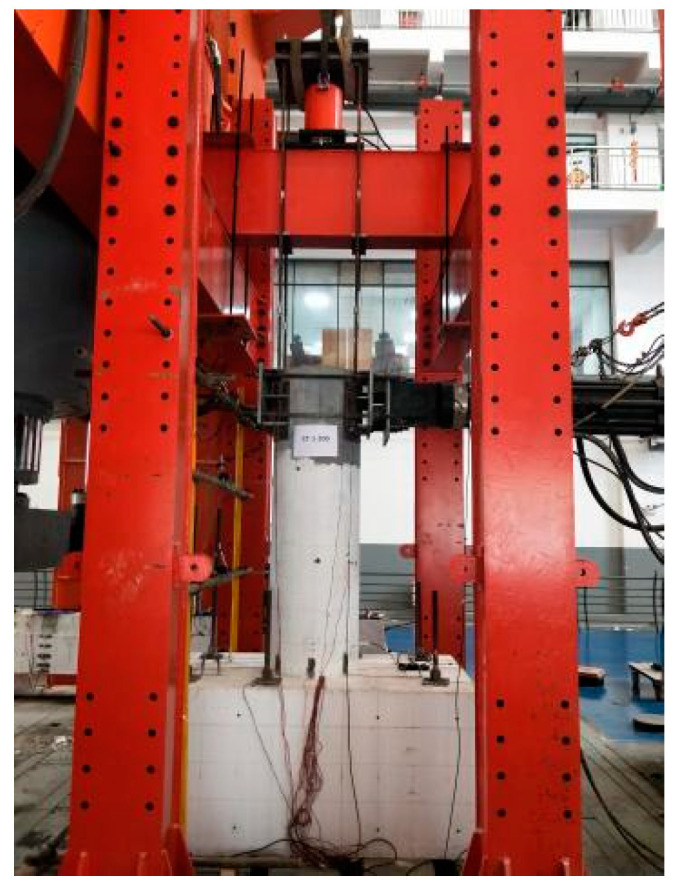
Physical drawing of loading device.

**Figure 3:**
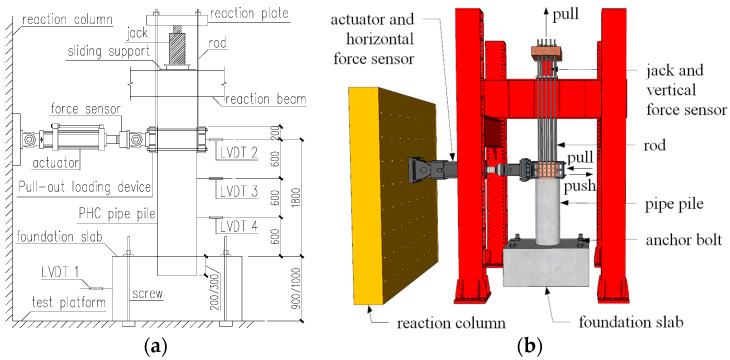
Schematic diagrams of the loading device. (**a**) Test Device Diagram 1; (**b**) Test Device Diagram 2.

The inverse position loading method was employed in the tests with the bearing platform located at the bottom of the pipe stake. The physical and schematic diagrams of the loading device are shown in Figure 2 and Figure 3. To measure the horizontal displacement of the stake body during the loading process, three displacement gauges were arranged along the length of the stake body. These resistance strain gauges were pasted on the anchoring reinforcement and pile concrete for measurement.

**Figure 4:**
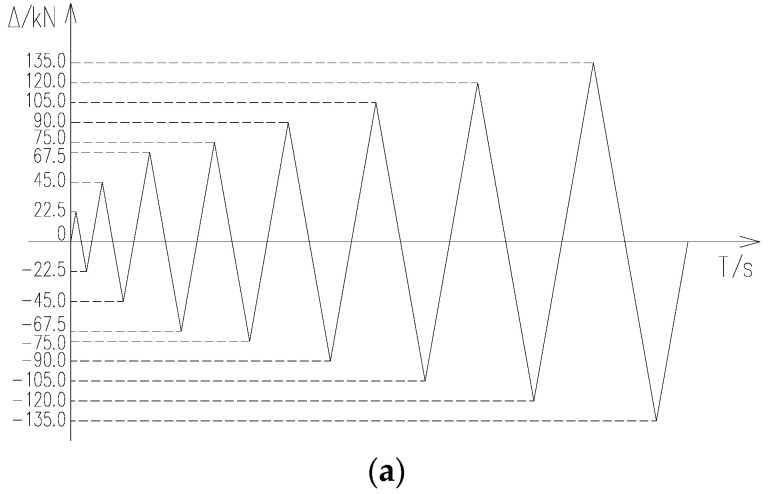

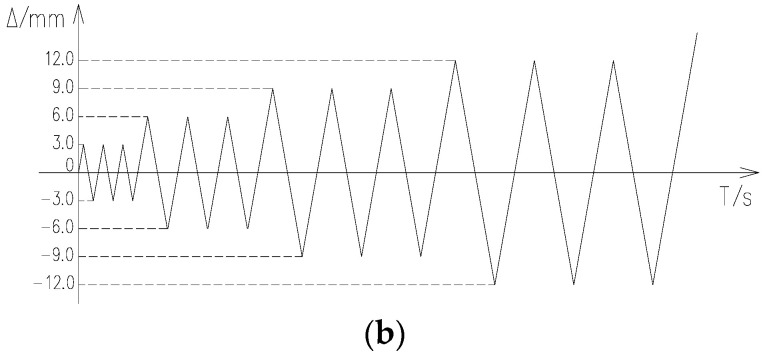
Specimen loading process diagram. (**a**) Force loading amplitude diagram; (**b**) displacement loading amplitude diagram.

During the test, the vertical 340 kN load was applied first, followed by the horizontal reciprocating load. The horizontal load was applied first with force and then with displacement [22]. The force was loaded once for each level of load reciprocation, and the displacement was loaded three times for each level of displacement reciprocation. The displacement loading amplitude was 3 mm. The loading process of the specimen is shown in Figure 4. The criterion for termination of the test is that the horizontal load is reduced to 85% of the ultimate load. During the test, the loading and unloading rate of each level of force was 1.0 kN/s, and the loading and unloading rate of each level of displacement was 120 s. The load holding time of each level was not less than 3 min.

During the test, the bearing platform is fixed with 6 30 mm diameter fine-rolled rebars with a tensile strength grade of 540 MPa, and the vertical prestressing force of each screw is 360 kN, totaling 2160 kN for the 6 screws, which is much larger than the vertical force of 340 kN. The bending moment that the screw can provide to the bearing platform is much larger compared to the bending moment that the horizontal load at the top of the stake can provide to the bearing platform. Therefore, it can be assumed that the bearing platform will not rotate.

Considering the horizontal movement of the bearing platform during the test, the horizontal displacement at the top of the stake is the difference between the horizontal displacement gauge at the top of the stake and the horizontal displacement gauge at the bearing platform.

## 3. Experimental Results

### 3.1. Appearance of Test

#### 3.1.1. Experimental Phenomena of CT-200

**Figure 5:**
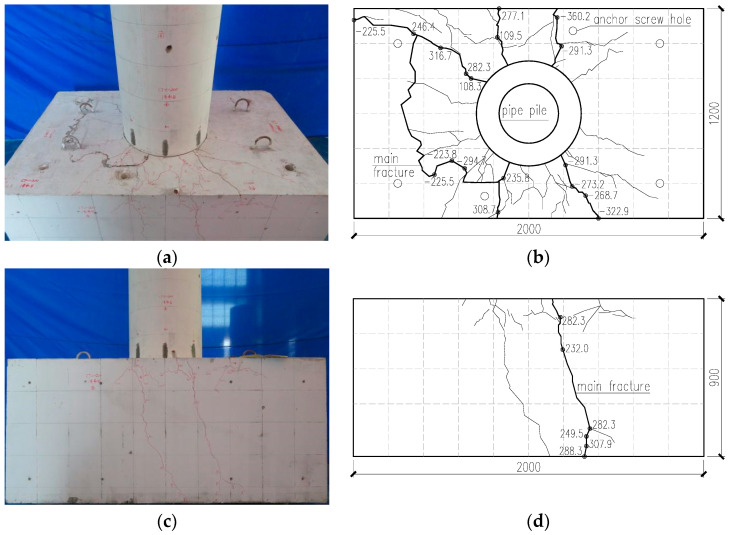
Damage to test CT-200. (**a**) Surface concrete damage on the cap; (**b**) cracks on the surface of the cap; (**c**) surface concrete damage on the south side of the cap; (**d**) cracks on the south side of the cap.

During the test, the concrete damage on the cap surface was very serious, and concrete cracks appeared on the whole cap. There were no visible cracks on the body of the pipe stake, which was not embedded into the cap. Figure 5 shows the damage to the test objects during the test process.

When a vertical tension force of 340 kN was applied to the top of the stake, a fine crack appeared at the connection between the pipe stake and the cap. When the force was loaded to ±135 kN, no radial cracks were observed in the bearing.

During the displacement loading process, when the actuator is loaded to the first circulation of 3.00 mm, the displacement of the top of the stake is 1.90 mm, the horizontal load is 108.30 kN, and the first crack appears on the surface of the cap, which is a radial crack centered on the center of the pipe stake.

As the displacement loading continues, cracks progressively develop on the surface of the cap and on the flanks of the cap on both sides of the loading direction. The length of the cracks extended and their width increased.

When the actuator is at 15.00 mm for the first circulation, the displacement of the top of the stake is 13.01 mm, and the horizontal load of the top of the stake is 308.70 kN. The widest crack on the surface of the cap is 0.85 mm, and the widest cracks on the south side and north side of the stake on both sides of the loading direction are 0.75 mm and 0.64 mm.

When the actuator is at −15.00 mm for the first circulation, the displacement of the top of the stake is −12.63 mm, and the horizontal load of the top of the stake is −348.80 kN. The widest crack on the surface of the cap is 0.69 mm, and the widest cracks on the south side and north side of the stake on both sides of the loading direction are 0.51 mm and 0.56 mm.

When the actuator is at 21.00 mm for the first circulation, the displacement of the stake top is 18.71 mm, and the horizontal load on the stake top is 327.10 kN. The width of the crack gap between the stake body and the bearing platform is 3.00 mm. When the actuator is at −21.00 mm for the first circulation, the displacement of the stake top is −18.68 mm, the horizontal load on the stake top is −363.10 kN, and the width of the crack on the surface of the bearing platform is 1.60 mm.

When the actuator is at −24.00 mm for the third circulation, the displacement of the top of the stake is −22.43 mm, and the horizontal load of the top of the stake is −306.70 kN. The widest crack on the surface of the bearing table is 1.99 mm, and the widest cracks on the south side and the north side of the stake on both sides of the loading direction are 0.82 mm and 0.89 mm.

When the actuator is at −27.00 mm for the third circulation, the displacement of the top of the stake is −26.07 mm, and the horizontal load of the top of the stake is −223.80 kN. The widest crack on the surface of the bearing table is 2.59 mm, and the widest cracks on the south side and the north side of the stake on the two sides of the loading direction are 1.56 mm and 1.34 mm.

When the actuator is in the first circulation of 39.00 mm, the displacement of the top of the stake is 35.66 mm, and the horizontal load of the top of the stake is 246.40 kN. The widest crack on the surface of the cap is 3.84 mm, and the widest cracks on the south side and north side of the stake on both sides of the loading direction are 1.50 mm and 1.41 mm. The width of the cracks between the stake body and cap is 7.0 mm.

In the late stage of the test, the concrete on the surface of the cap was obviously bulging, and the sound of a hollow drum was obvious when knocking.

#### 3.1.2. Experimental Phenomena of CT-300

**Figure 6:**
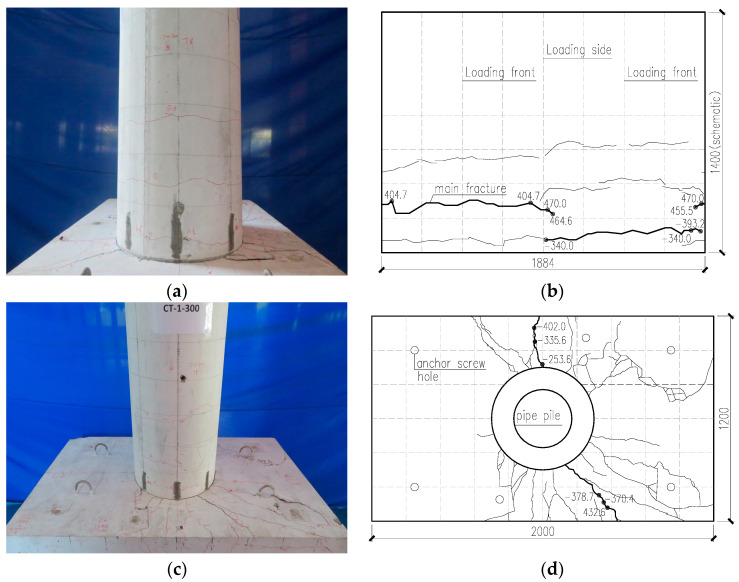
Damage to test CT-300. (**a**) Failure pattern of the pipe pile; (**b**) pipe pile cracking diagram; (**c**) surface concrete damage on the cap; (**d**) cracks on the surface of the cap.

During the loading process, both the pipe stake concrete and cap concrete showed obvious cracking. Figure 6 shows the cracking of concrete.

When a vertical tension force of 340 kN was applied to the top of the stake, a fine crack appeared at the connection between the pipe stake and the cap. When the force was loaded to −120 kN, the surface ring crack penetrated at the contact between the stake and the cap. During force loading, no radial cracks were observed in the bearing platform when the force was ±135 kN.

During the displacement loading process, when the actuator is loaded to the first circulation of 3.00 mm, the displacement of the top of the stake is 2.09 mm, the horizontal load is 178.50 kN, and the first crack appears on the surface of the cap, which is a radial crack centered on the center of the pipe stake.

When the actuator is at 9.00 mm for the first circulation, the displacement of the top of the stake is 7.57 mm, and the horizontal load of the top of the stake is 404.70 kN. Ring crack began to appear in the stake. As loading proceeds, the number of circumferential cracks in the pile body increases, and the cracks become longer.

When the actuator is at 15.00 mm for the third circulation, the displacement of the top of the stake is 12.93 mm, and the horizontal load of the top of the stake is 408.50 kN. The widest crack in the stake was 0.35 mm. The widest crack on the surface of the cap is 0.66 mm, and the widest cracks on the south side and the north side of the stake on both sides of the loading direction are 0.49 mm and 0.29 mm.

When the actuator is at −15.00 mm for the third circulation, the displacement of the top of the stake is −12.48 mm, and the horizontal load of the top of the stake is −365.80 kN. The widest crack in the stake was 0.33 mm. The widest crack on the surface of the cap is 0.60 mm, and the widest cracks on the south side and the north side of the stake on both sides of the loading direction are 0.47 mm and 0.33 mm.

When the actuator is at −21.00 mm for the third circulation, the displacement of the top of the stake is −17.90 mm, and the horizontal load of the top of the stake is −420.10 kN. The widest crack in the stake was 0.39 mm. The widest crack on the surface of the cap is 1.13 mm, and the widest cracks on the south side and the north side of the stake on both sides of the loading direction are 0.38 mm and 0.64 mm.

When the actuator is at −24.00 mm for the second circulation, the displacement of the top of the stake is −20.74 mm, and the horizontal load of the top of the stake is −445.70 kN. The widest crack in the stake was 0.41 mm. The widest crack on the surface of the cap is 1.31 mm, and the widest cracks on the south side and the north side of the stake on both sides of the loading direction are 1.13 mm and 0.50 mm. At this time, the concrete on the surface of the cap has no warping visible, but the sound of the hollow drum can be heard when knocking.

When the actuator is at 27.00 mm for the first circulation, the displacement of the top of the stake is 24.21 mm, and the horizontal load of the top of the stake is 481.60 kN. The widest crack in the stake was 0.45 mm. The widest crack on the surface of the cap is 1.37 mm, and the widest cracks on the south side and the north side of the stake on both sides of the loading direction are 0.42 mm and 1.92 mm.

When the actuator is at −30.00 mm for the third circulation, the displacement of the top of the stake is −27.83 mm, and the horizontal load of the top of the stake is −453.10 kN. The widest crack in the stake was 0.45 mm. The widest crack on the surface of the cap is 2.00 mm, and the widest cracks on the south side and the north side of the stake on both sides of the loading direction are 0.94 mm and 3.23 mm.

When the actuator is at 33.00 mm for the third circulation, the displacement of the top of the stake is 29.18 mm, and the horizontal load of the top of the stake is 389.00 kN. The widest crack in the stake was 0.36 mm. The widest crack on the surface of the cap is 2.31 mm, and the widest cracks on the south side and the north side of the stake on both sides of the loading direction are 1.00 mm and 2.52 mm.

When the actuator is at −33.00 mm for the third circulation, the displacement of the top of the stake is −31.14 mm, and the horizontal load of the top of the stake is −424.20 kN. The widest crack in the stake was 0.37 mm. The widest crack on the surface of the cap is 2.75 mm, and the widest cracks on the south side and the north side of the stake on both sides of the loading direction are 1.41 mm and 1.61 mm.

When the actuator is at −39.00 mm for the third circulation, the displacement of the top of the stake is −37.67 mm, and the horizontal load of the top of the stake is −393.20 kN. The widest crack in the stake was 0.35 mm. The widest crack on the surface of the cap is 4.20 mm, and the widest cracks on the south side and the north side of the stake on both sides of the loading direction are 2.24 mm and 1.50 mm.

In the late stage of the test, as in the JCT-200 specimen, the concrete on the bearing surface buckled and damaged.

For the two specimens with embedment depths of 200 mm and 300 mm, the bearing capacity of the bearing node gradually decreased after the destruction of the bearing concrete in contact with the stake. After the destruction of the node, the anchoring reinforcement in the node area yielded, the restraint was weakened, an articulated node was formed, the rotational capacity was enhanced, and the concrete on the surface of the bearing platform buckled and cracked.

### 3.2. Strain in Anchored Reinforcement

**Figure 7:**
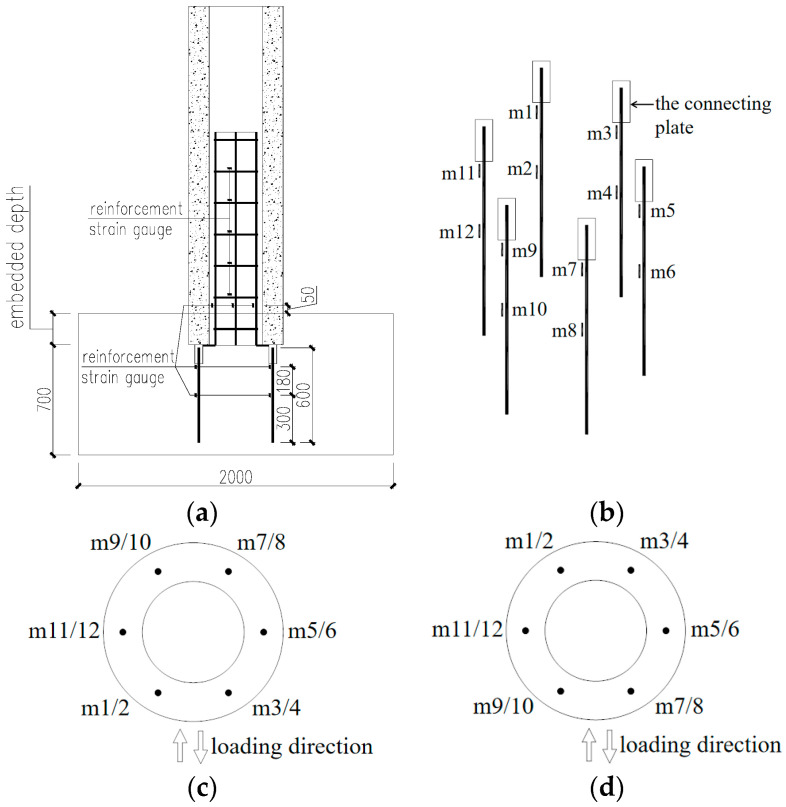
Location of strain gauges for reinforcement. (**a**) Location of strain gauges for specimen reinforcement; (**b**) Anchor Reinforcement Strain Gauge Location 1; (**c**) Anchor Reinforcement Strain Gauge Location 2; (**d**) Anchor Reinforcement Strain Gauge Location 3.

During the tests, strain gauges were posted on the anchored and core-filled bars to measure the rebar strains. The locations of the rebar strain gauges are shown in Figure 7.

#### 3.2.1. Strain in Anchored Reinforcement for CT-200

**Figure 8:**
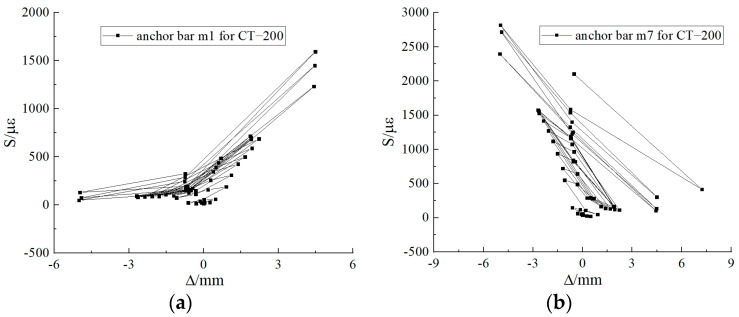
Anchored steel strain gauge for CT-200. (**a**) Strain diagram for Anchoring Reinforcement m1; (**b**) Strain diagram for Anchoring Reinforcement m7.

The location of the anchoring reinforcement in CT-200 is shown in Figure 7c. Throughout the loading process, the anchoring reinforcement yielded and plastic deformation occurred. During the test, the strain gauges m1 and m7 of the anchoring reinforcement were larger. The corresponding top pile displacement was 7.22 mm and the top pile horizontal load was 282.30 kN when the anchoring bar m1 yielded, and the corresponding top pile displacement was −5.01 mm and the top pile horizontal load was −246.50 kN when the anchoring bar m7 yielded. When the specimen was damaged, the maximum strain of the anchoring bar m1 was close to 55,000 με, and that of the anchoring bar m7 was close to 60,000 με. The strains in the anchored reinforcement before the strain surge are shown in Figure 8.

#### 3.2.2. Strain in Anchored Reinforcement for CT-300

**Figure 9:**
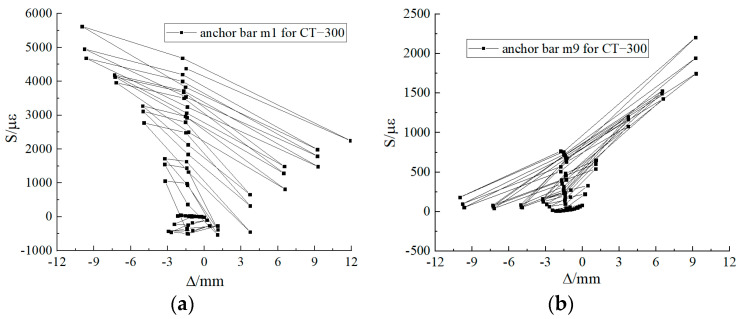
Anchored steel strain gauge for CT-300. (**a**) Strain diagram for Anchoring Reinforcement m1; (**b**) strain diagram for Anchoring Reinforcement m9.

The location of anchoring reinforcement in CT-300 is shown in Figure 7d. Throughout the loading process, the anchoring reinforcement yielded and plastic deformation occurred. During the test, the strain gauges m1 and m9 of the anchoring reinforcement were larger. The corresponding pile top displacement when Anchoring Bar m1 yielded was −3.93 mm, and the horizontal load at the pile top was −255.90 kN; the corresponding pile top displacement when the Anchoring Bar m9 yielded was 10.22 mm, and the horizontal load at the pile top was 445.60 kN. When the specimen was damaged, the maximum strain of Anchoring Bar m1 was close to 75,000 με, and that of Anchoring Bar m9 was close to 60,000 με. The strains of the anchorage bars before the strain surge are shown in Figure 9.

### 3.3. Skeleton Curve

**Figure 10:**
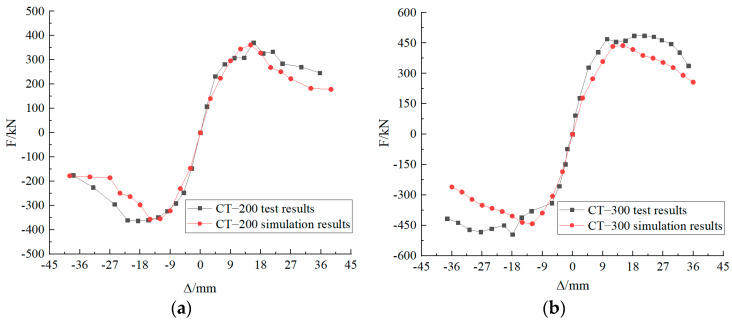
Load–displacement skeleton curves. (**a**) CT-200 skeleton curves; (**b**) CT-300 skeleton curves.

Figure 10 shows the load–displacement skeleton curves for CT-200 and CT-300 test objects.

From the load-top displacement skeleton curve of the specimen in Figure 10, it can be seen that in the initial stage, the nodal load–displacement relationship curve is linear and the specimen is elastic. As the loading proceeds, the specimen is in the elastic–plastic stage, and the concrete of the stake and cap gradually cracks until the ultimate bearing capacity is reached. After reaching the ultimate load-carrying capacity, the specimen enters the plastic stage and rapidly loses the load-carrying capacity.

## 4. Numerical Simulation

### 4.1. Numerical Model

Finite element simulation can overcome the shortcomings of the insufficient number of foot-scale tests. The geometric and physical parameters of the finite element model in this paper are exactly the same as those of the test objects.

An automatic incremental step is used in the simulation process, and the nonlinear switch is turned on. There are four analysis steps: the first step uses the cooling method to apply prestressing force, the second step applies 340 kN vertical force, the third step applies horizontal load, and the fourth step applies horizontal displacement.

The CDP model given in [23] is a widely applied and mature method, so this paper adopts the formula method in the code. The expansion angle is taken as 30°, eccentricity is taken as 0.1, the ratio of biaxial compressive strength to uniaxial compressive strength is taken as 1.16, the ratio of second stress invariant on the tensile meridian is taken as 0.6667, and the coefficient of viscosity is taken as 0.001. The concrete density is taken as 2500 kg/m^3^, and Poisson’s ratio is taken as 0.2. The modulus of elasticity of concrete with compressive strengths of 38.71 MPa, 40.30 MPa, and 93.20 MPa were 32,166.0 MPa, 32,601.0 MPa, and 41,004.0 MPa, respectively. The compression recovery for concrete was set at 0.8.

In order to make the simulation close to the test conditions, fixed boundary conditions were used for 200 mm at both ends of the cap.

The stake is in bound contact with the core filler. Coulomb contact is used between the stake and the cap. Reinforcing steel is built into the whole model. The bearing concrete and core-fill concrete are modeled as a whole.

**Figure 11:**
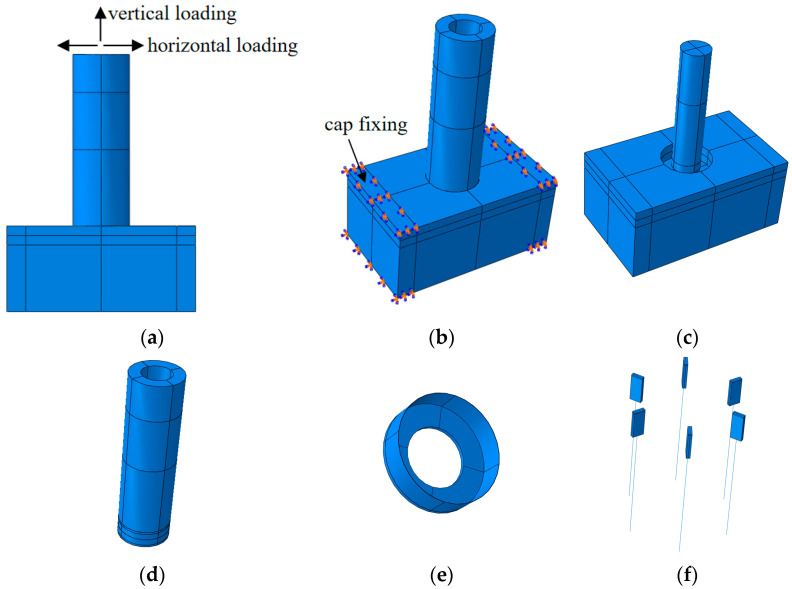

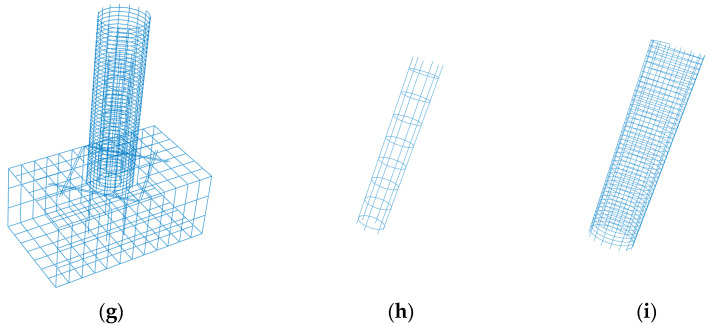
The components of the finite element model. (**a**) Loading schematic; (**b**) bearing platform fixation; (**c**) bearing and core filling; (**d**) PHC stake; (**e**) terminal plate and pile ferrule; (**f**) connection plates and anchoring bars; (**g**) reinforcement; (**h**) core filling steel; (**i**) pile reinforcement.

In modeling, each component is modeled in a separated manner. The solid parts are modeled using C3D8R units and the reinforcement bars are modeled using T3D2 truss units. The components of the finite element model are shown in Figure 11.

A non-prestressed component such as a bearing cap was deactivated during the application of prestressing.

**Figure 12:**
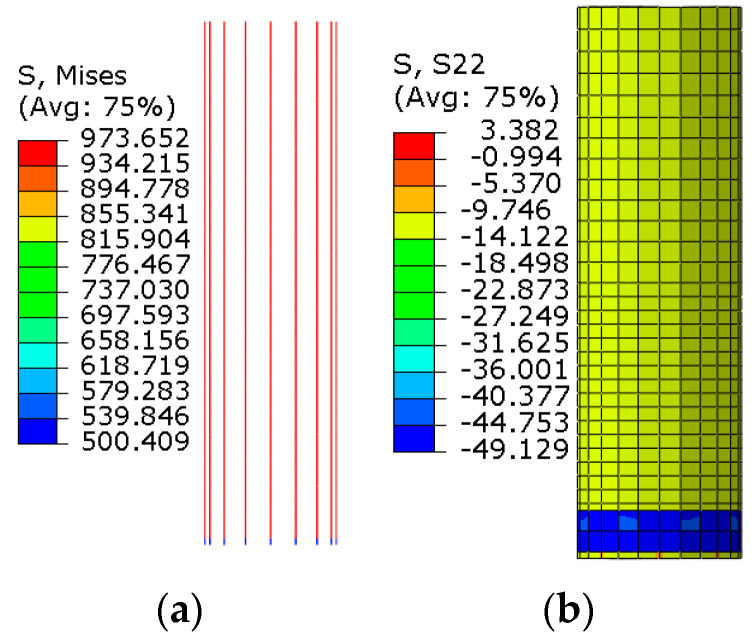
The stress cloud. (**a**) Stress cloud diagram of reinforcement; (**b**) stress cloud diagram of stake concrete.

The stress cloud of the longitudinal reinforcement of the tubular stake and the stake concrete after applying prestressing is shown in Figure 12. The longitudinal reinforcement prestressing of the tubular pile is about 973.70 MPa, and the concrete prestressing in the middle of the pile is about 10.18 MPa.

### 4.2. Numerical Simulation Validation

The material properties parameters in the numerical simulation are the same as the test material properties parameters, as described in Section 2.1.

#### 4.2.1. Comparison of Simulated and Experimental Skeleton Curves

(1) Reinforced concrete

**Figure 13:**
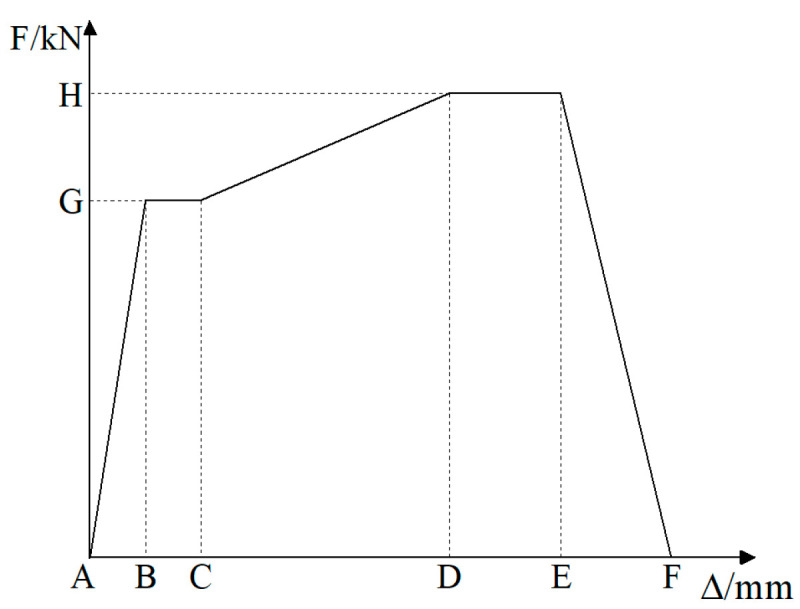
Pentagonal model.

**Figure 14:**
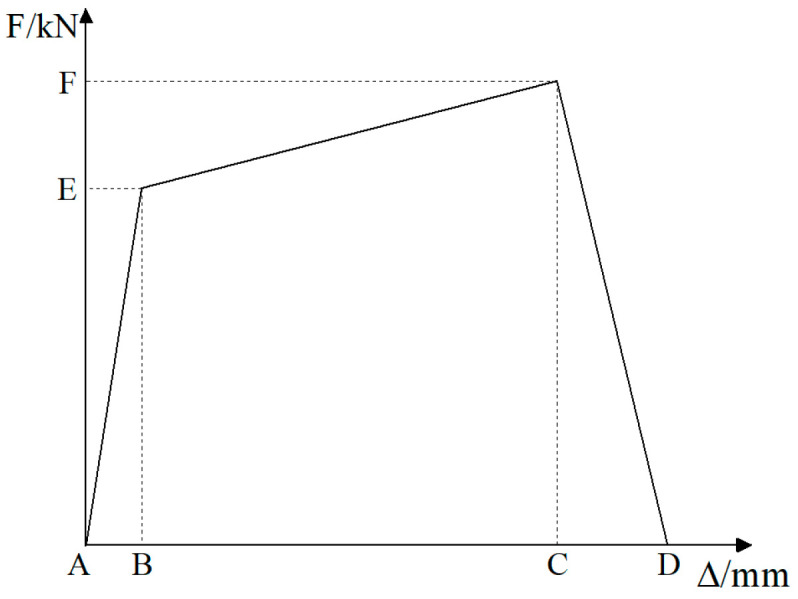
Trifold model.

According to the test material properties data in Table 2, a five-fold model was used in the numerical simulation for bars with yielding stages. For the rebars without obvious yielding stage, the three-fold model was used, as detailed in Figure 13 and Figure 14.

During the test, the density of the steel was 7800 kg/m^3^, the Poisson’s ratio was 0.3, and the rest of the mechanical parameters are shown in Table 3.

(2) Concrete substrate

**Figure 15:**
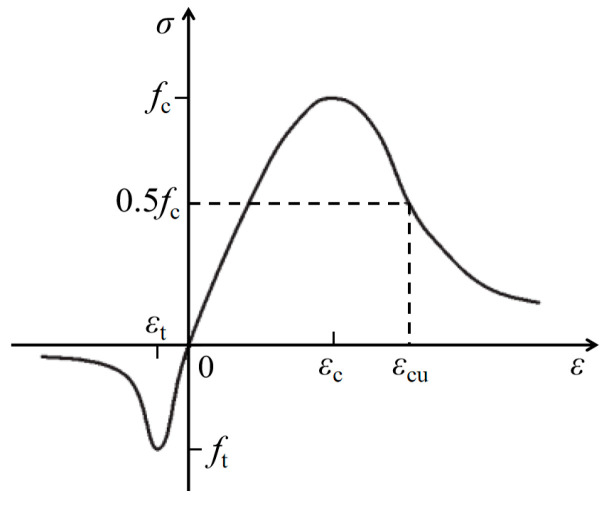
Concrete substrate.

Figure 15 shows the stress–strain curves of concrete in uniaxial tension and compression [23].

Equations (1)–(4) illustrate the stress–strain relationship for concrete subjected to axial tension.

(1) $$\sigma =(1-d_{t})E_{c}\varepsilon$$

(2) $$d_{t}=\left\{ \begin{array}{l} 1-\rho_{t}[1.2-0.2x^{5}]\;\;\;\;x\leq 1\\ 1-\frac{\rho_{t}}{a_{t}{\left(x-1\right)}^{1.7}+x}\;\;\;\;\;\;\;\;\;\;\;x>1 \end{array}\right.$$

(3) $$x=\frac{\varepsilon }{\varepsilon_{t,r}}$$

(4) $$\rho_{t}=\frac{f_{t,r}}{E_{c}\varepsilon_{t,r}}$$

The parameter “*d*_t_” represents the uniaxial tensile damage evolution of concrete. The parameter “*E*_c_” denotes the modulus of elasticity of concrete. The parameter “*f*_t,r_” signifies the representative value of the uniaxial tensile strength of concrete. The parameter “*ε*_t,r_” indicates the peak tensile strain of concrete corresponding to *f*_t,r_. The parameter “*a*_t_” specifies the value of the descending section of the uniaxial tensile stress–strain curve of concrete.

Equations (5)–(9) illustrate the stress–strain relationship for concrete subjected to axial compression.

(5) $$\sigma =(1-d_{c})E_{c}\varepsilon$$

(6) $$d_{c}=\left\{ \begin{array}{l} 1-\frac{\rho_{c}n}{n-1+x^{n}}\;\;\;\;\;\;\;\;\;x\leq 1\\ 1-\frac{\rho_{c}}{a_{c}{\left(x-1\right)}^{2}+x}\;\;\;\;x>1 \end{array}\right.$$

(7) $$\rho_{c}=\frac{f_{c,r}}{E_{c}\varepsilon_{c,r}}$$

(8) $$n=\frac{E_{c}\varepsilon_{c,r}}{E_{c}\varepsilon_{c,r}-f_{c,r}}$$

(9) $$x=\frac{\varepsilon }{\varepsilon_{c,r}}$$

The parameter “*d*_c_” represents the uniaxial compressive damage evolution of concrete. The parameter “*f*_c,r_” signifies the representative value of the uniaxial compressive strength of concrete. The parameter “*ε*_c,r_” indicates the peak compressive strain of concrete corresponding to *f*_c,r_. The parameter “*a*_c_” specifies the value of the descending section of the uniaxial compressive stress–strain curve of concrete.

(3) Numerical simulation validation

In the model validation process, the loading scheme of the numerical simulation is identical to that of the test, in which the horizontal direction is subjected to a combination of force and displacement. The comparison of the skeleton curves for model validation is presented in Figure 10. From Figure 10, it can be observed that the simulated skeleton curves and the test skeleton curves are in close agreement, thereby demonstrating the reliability of the numerical simulation.

#### 4.2.2. Comparative Analysis of Nodal Load Capacity

A comparison of the results of the numerical simulation of the nodal load capacity with the results of the test of the nodal load capacity is presented in Table 4.

As illustrated in Table 4, an increase in the depth of node embedment from 200 mm to 300 mm resulted in a 31.04% enhancement in the ultimate shear force and bending moment of the specimen in the forward direction and a 36.16% augmentation in the ultimate shear force and bending moment in the reverse direction. The ultimate bending moment is 1.8 times the ultimate shear force, given that the top of the stake is 1.8 m from the bearing surface.

The numerical simulated nodal bearing capacity of CT-200 is 0.98 times the test value, while the numerical simulated nodal bearing capacity of CT-300 is 0.90 times the test value. Similarly, the numerically simulated stake-top horizontal displacement of CT-200 is 0.88 times the test value, while the numerically simulated stake-top horizontal displacement of CT-300 is 0.69 times the test value. In general, the numerical simulations are accurate.

#### 4.2.3. Nodal Concrete Damage

**Figure 16:**
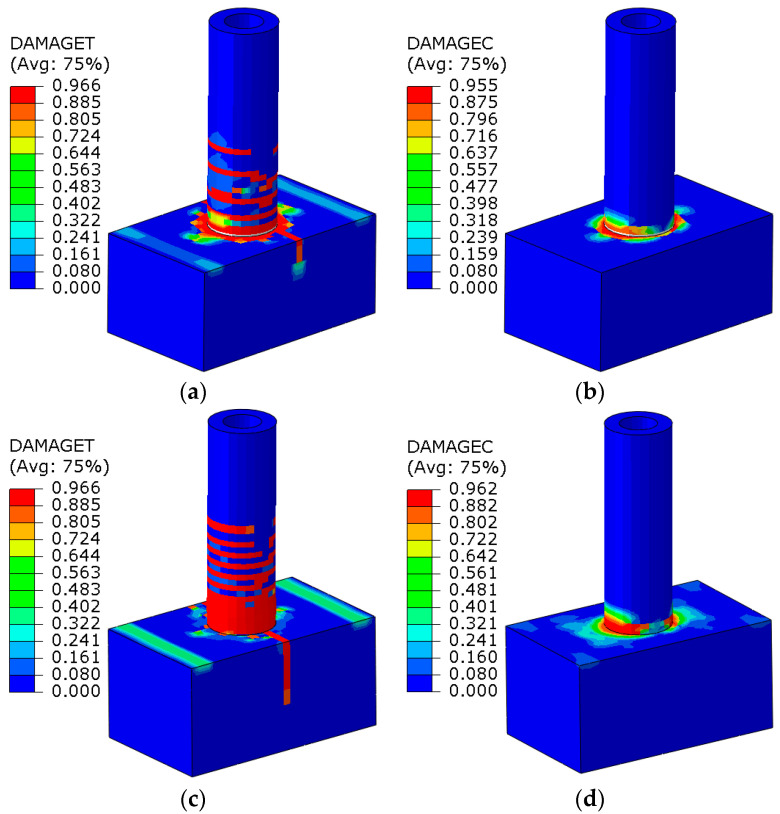
Numerically simulated concrete damage cloud. (**a**) CT-200 nodal concrete tensile damage; (**b**) CT-200 nodal concrete compression damage; (**c**) CT-300 nodal concrete tensile damage; (**d**) CT-300 nodal concrete compression damage.

Figure 16 depicts the numerically simulated concrete damage cloud.

As illustrated in Figure 16, the tensile damage to both the stake body and cap is greater than the compression damage. Furthermore, the stake body remains undamaged throughout the loading process. In comparison to an embedment depth of 200 mm, the tensile damage of the stake body is more pronounced at an embedment depth of 300 mm. These observations align well with the test results.

### 4.3. Bearing Capacity of Four Types of Nodes

In the test and numerical simulation verification process, both are Type I nodes, and the material property parameters utilized in the simulation are identical to those employed in the test.

Due to the specificity of the test material properties, in order to generalize the simulation results, the material properties shown in Table 1 are used in the numerical analysis of Type I, Type II, Type III, and Type IV nodes in the following sections instead of the test parameters. Furthermore, the modeling method is the same as that described in Section 4.1.

#### 4.3.1. Material Strength

The bearing and core filling were constructed using C30 concrete, and the stake was constructed using C80 concrete. The modulus of elasticity of concrete with compressive strengths of 30.0 MPa and 80.0 MPa were 30,000.0 MPa and 38,000.0 MPa, respectively. The precompression stress of the stake concrete was 8.40 MPa.

The terminal plate and connecting plate are made of Q235B steel, and the pile ferrule is made of Q235 steel [21]. The rebar is modeled using a bifold model comprising an elastic section and a reinforcement section. Prior to yielding, the rebar exhibits perfect elastic behavior. After yielding, the stress–strain relationship is simplified to a highly smooth inclined straight line. Additionally, the Young’s modulus of the rebar after yielding is 0.01 times that of the Young’s modulus before yielding [24].

As outlined in the “Prestressed Concrete Pile” [21], the specified nonproportional elongation strength of prestressed steel rods should not be less than 1280 MPa, and the standard tensile strength should not be less than 1420 MPa. In accordance with Article 6.4 of the “Cold-Drawn Low-Carbon Steel Wire for Concrete Products” [25], the tensile strength of cold-drawn low-carbon steel wire must exceed 550 MPa, while the elongation at break must exceed 2.0%. The ABAQUS models of the physico-mechanical parameters of the steel rods and hoops are presented in Table 5.

#### 4.3.2. Bearing Capacity of the Terminal Plate Welded Anchor Bar + Core-Filled Longitudinal Bar Unanchored into the Cap Node (Type I Node)

Core-filled longitudinal bars were anchored in the load-bearing nodes of the specimens with embedment depths ranging from 50 to 500 mm. Table 6 shows the results of the numerical simulation of a Type I node.

As illustrated in Table 6, for a Type I node, the embedment depth of 250 mm (0.42 times the stake outer diameter D) or greater results in a relatively constant bearing capacity. It can thus be posited that damage to the node is caused by damage to the stake body once the embedment depth exceeds 250 mm. In consideration of the node-bearing capacity, the recommended embedment depth is 250 mm.

#### 4.3.3. Bearing Capacity of the Terminal Plate Welded Anchor Bar + Core-Filled Longitudinal Bar Anchored into the Cap Node (Type II Node)

The Type II node is a pipe pile terminal plate welded anchor bar + core-filled longitudinal bar anchored into the cap node. The finite element simulation results of the bearing capacity of the stake anchored to the cap node are listed in Table 6.

As illustrated in Table 6, when the embedment depth is less than or equal to 200 mm (0.33 D), the ultimate bearing capacity of the Type II node is observed to be greater than that of the Type I node. When the embedment depth is greater than or equal to 250 mm (0.42 D), the ultimate bearing capacity of the Type II node is comparable to that of the Type I node and essentially unaltered. It can thus be posited that damage to the node is a consequence of damage to the stake once the embedment depth exceeds 250 mm. In light of the aforementioned findings, the recommended embedment depth is 250 mm, in accordance with the node-bearing capacity.

#### 4.3.4. Bearing Capacity of the Core-Filled Longitudinal Bar Anchored into the Cap Node (Type III Node)

The Type III node is the node with core-filled longitudinal reinforcement anchored into the cap.

Table 6 illustrates that when the embedment depth is equal to or less than 300 mm (0.50 D), the ultimate bearing capacity of Type III nodes is less than that of Type I and Type II nodes. At embedment depths exceeding 350 mm (0.58 D), the ultimate load-carrying capacity of Type III nodes is comparable to that of Type I and Type II nodes and exhibits minimal variation. Consequently, damage to the nodes at embedment depths greater than 350 mm is considered to be caused by damage to the stake. The recommended embedment depth is 350 mm, based on the load-carrying capacity of the nodes.

#### 4.3.5. Bearing Capacity of the Terminal Plate Welded Anchor Bar Node (Type IV Node)

Type IV nodes are terminal plate welded anchorage reinforcement nodes.

As illustrated in Table 6, the ultimate bearing capacity of Type IV nodes is consistently inferior to that of Type I and Type II nodes throughout the loading process. When the embedment depth is less than or equal to 250 mm (0.42 D), the ultimate bearing capacity of Type IV nodes is greater than that of Type III nodes. The ultimate load capacity of Type IV nodes is less than that of Type III nodes at depths of burial greater than or equal to 300 mm (0.50 D).

The ultimate bearing capacity of Type IV nodes remains essentially unchanged at embedment depths greater than or equal to 200 mm (0.33 D), indicating that damage to Type IV nodes beyond this depth is primarily caused by damage to the stake. It can thus be concluded that damage to the node is caused by damage to the stake at embedment depths greater than 200 mm. The maximum load-carrying capacity of Type IV nodes is less than that of Type I and Type II nodes. Consequently, the use of Type IV nodes is not advised.

The maximum load-carrying capacity of Type I, Type II, and Type III nodes is comparable, with average values of approximately 717.94 kN·m and 719.14 kN·m, respectively. In contrast, the load-carrying capacity of Type IV nodes is approximately 565.74 kN·m and 574.25 kN·m, respectively. The impact of reinforced concrete infill on the node load-carrying capacity is notable, exhibiting a positive growth rate of approximately 26.90% and a negative growth rate of about 25.23%. The effect of reinforced concrete infill on the node load-carrying capacity is significant, with a positive growth rate of approximately 26.90% and a negative growth rate of approximately 25.23%.

When the vertical force is 340 kN, the embedment depths of the Type I and Type II nodes are the same at the same ultimate bearing capacities. However, the terminal plates of Type II nodes are welded and anchored to the bearing table, and the vertical tensile capacity of Type II nodes is greater than that of Type I nodes. Compared with Type III nodes, Type II nodes have a smaller embedding depth when they reach the ultimate bearing capacity. And the construction of a Type II node is convenient. Therefore, in engineering practice, the use of Type II nodes is recommended, and the embedment depth is 250 mm. Simultaneously, the welding quality between the connecting plate, the anchorage bar of the terminal plate, and the terminal plate should be strictly guaranteed in the project.

**Figure 17:**
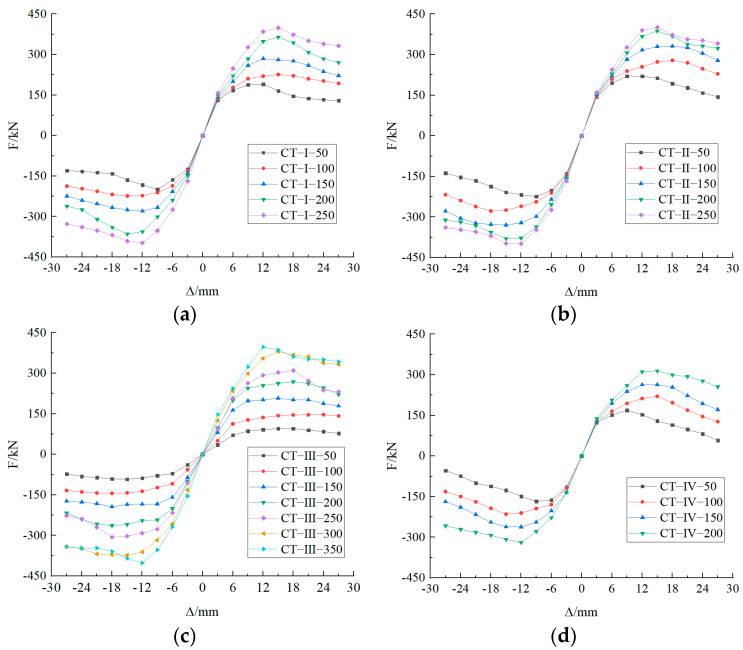
Skeleton curves of Type I, Type II, Type III, and Type IV nodes. (**a**)Type I node skeleton curve; (**b**) Type II node skeleton curve; (**c**) Type III node skeleton curve; (**d**) Type IV node skeleton curve.

In Clause 4.4.4 of [22], the value of bearing capacity reduction should be regulated to 85% of the ultimate load. In the simulation, when the displacement is ±27 mm, the bearing capacity of the specimen is observed to decrease to 85% of the ultimate load. Figure 17 illustrates the skeleton curves of nodes of Types I, II, III, and IV, which are less than or equal to the optimal embedment depth.

As illustrated in Figure 17, the impact of embedment depth on the initial stiffness of Type I, Type II, and Type IV nodes is relatively modest. The effect on the initial stiffness of Type III nodes is more pronounced.

#### 4.3.6. Bearing Capacity under Different Vertical Tensile Forces

Due to the use of 6 HRB400 steel bars with a longitudinal bar diameter of 18 mm, the maximum tensile force that the steel bars can withstand before yielding is 610.42 kN. The terminal plate welded anchor bars are 6 HRB 400 steel bars with a diameter of 20 mm, and the maximum tensile force that the steel bars can withstand before yielding is 753.60 kN. As indicated in reference [21], the design value of the axial tensile capacity of PHC B 600-130 stake bodies is 1700 kN. Consequently, the vertical force employed in this study ranges from 0 to 1360 kN. Table 7 illustrates the finite element calculation outcomes for the bearing capacity of the nodes under disparate vertical tensile forces.

**Figure 18:**
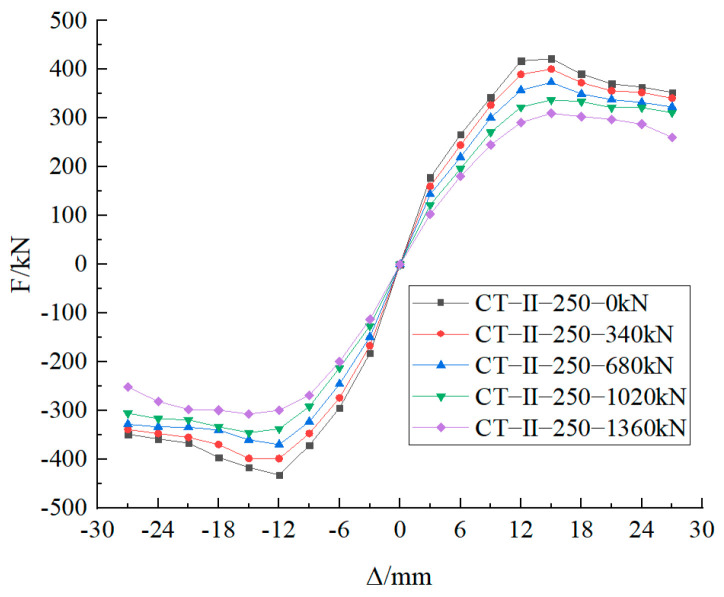
Skeleton curves of Type II nodes with optimal embedment depth.

Figure 18 illustrates the skeletal curves of Type II nodes under varying vertical tensile forces.

As illustrated in Table 7 and Figure 18, following the Type II node attains the optimal embedment depth, the bearing capacity of the node declines with rising vertical tension. However, the displacement corresponding to the ultimate bearing capacity remains largely unaltered. When the vertical tension ascends from 0 to 1360 kN, the positive and negative bearing capacities of Type II nodes diminish by 26.61% and 28.97%, respectively.

## 5. Conclusions

In this paper, low-cycle reciprocating load tests were conducted on two nodes connecting PHC piles and caps. The test phenomena and node-bearing capacity were then analyzed. The finite element method was employed to compare the bearing capacity of various node types and embedment depths, with the objective of identifying the optimal node type and optimal embedment depth. The principal conclusions are as follows:

(1) The test results indicate that when the embedment depth is 200 mm and 300 mm, the cap concrete in the node area experiences buckling damage, the anchoring reinforcement yields, the constraint is weakened, the articulation point is formed, and the node rotational capacity increases. The embedment depth was increased from 200 mm to 300 mm, resulting in an increase in the ultimate bearing capacity of the node in both the forward and reverse directions, by 31.04% and 36.16%, respectively.
(2) The finite element calculation results show that the maximum load-carrying capacity of the terminal plate welded anchor bar + core-filled longitudinal bar unanchored into the cap node (Type I node), the terminal plate welded anchor bar + core-filled longitudinal bar anchored into the cap node (Type II node), and the core-filled longitudinal bar anchored into the cap node (Type III node) are close to each other, with average values of approximately 717.94 kN·m and −719.14 kN·m, respectively. The bearing capacities of the terminal plate welded anchor bar nodes (Type IV nodes) are approximately 565.74 kN·m and −574.25 kN·m, respectively. The positive increase in bearing capacity of the bearing nodes after core filling is about 26.90% and the negative increase is about 25.23% as compared to the nodes without core filling.
(3) It is recommended to use a Type II node, whose embedment depth is 250 mm (0.42D). It should be noted that this numerical simulation does not account for the error generated by the slip between the reinforcement and the concrete structure. As a result, the simulated pile-top displacement is generally smaller than the actual pile-top displacement. Therefore, it is appropriate to increase the embedment depth in the project.
(4) When the optimal embedding depth of Type II nodes is 250 mm and the vertical tension increases from 0 to 1360 kN, the positive and negative bearing capacities of Type II nodes decrease by 26.61% and 28.97%, respectively.

## Figures and Tables

**Table 1:** Parameters of the specimens.

Specimen Number	Embedding Depth/mm	Prestressing Longitudinal Tendons	Pipe Pile Hoop	Prestressing of Tubular Piles before Pile Cutting/MPa	Terminal Plate Welded Anchorage Bar (HRB400)	Anchoring Bar Length/mm	Terminal Plate Welded Anchorage Bar Distribution Circle Diameter (mm)	Filling Longitudinal Bar (HRB400)	Filling Longitudinal Bar Distribution Circle Diameter (mm)
CT-200	200	16φ12.6	Φ^b^5	8.40	6@20	600	470	6@18	260
CT-300	300								

**Table 2:** Rebar properties of the test objects.

Designation	Type	Diameter/mm	Yield Strength/MPa	Elastic Modulus/GPa	Yield Point Elongation/%	Tensile Strength/MPa	Maximum Force Plastic Elongation/%	Maximal Load Stretching/%	Maximum Force Elongation/%	Percentage Elongation after Fracture/%
Pile stirrups	—	5.0	523.48	200.13	—	595.33	2.56	4.29	—	5.30
Prestressed steel rod of pile body	—	12.6	1370.61	227.65	—	1471.94	4.05	5.40	—	7.88
Filling core stirrups	HPB300	8.0	356.42	210.22	2.95	540.34	20.80	22.74	4.31	25.85
Pile cap reinforcement	HRB335	14.0	547.88	209.60	2.49	618.68	6.60	7.75	1.47	15.33
Filling core anchorage steel bar	HRB400	18.0	456.25	206.82	3.83	619.85	15.67	16.92	6.03	24.24
Terminal plate welded anchorage bar	HRB400	20.0	436.17	204.15	2.57	621.56	16.70	18.01	6.33	26.12

**Table 3:** Mechanical parameters of steel used in the test.

Designation	Model Number	Caliber/mm	Young’s Modulus/MPa	Plastic	
				Yield Strength/MPa	Plastic Strain
Pile hoop	—	5.0	200,130	523.48	0
				595.33	0.026
Pile prestressing steel rods	—	12.6	227,650	1370.61	0
				1471.94	0.041
				981.30	0.078
Core-filling hoop	HPB300	8.0	210,220	356.42	0
				356.42	0.030
				540.34	0.208
				540.34	0.251
Bearing reinforcement	HRB335	14.0	209,600	547.88	0
				547.88	0.025
				618.68	0.066
				618.68	0.081
Core-filled reinforcement	HRB400	18.0	206,820	456.25	0
				456.25	0.038
				619.85	0.157
				619.85	0.217
				465.00	0.241
Terminal plate welded anchorage bar	HRB400	20.0	204,150	436.17	0
				436.17	0.026
				621.56	0.193
				621.56	0.256
				466.00	0.260

**Table 4:** A comparative analysis of the finite element calculation results for specimen bearing capacity with the corresponding test results.

Specimen Number	Load Direction	Test Limit Load/kN	Test Ultimate Bending Moment/kN·m	Displacement of the Loaded End Corresponding to the Ultimate Load/mm	Simulated Ultimate Loads/kN	Simulation of Ultimate Bending Moment/kN·m	Simulation of Limit Displacements/mm	Calculated Value of Ultimate Load/Experimental Value	Calculated Value of Ultimate Displacement/Experimental Value
CT-200	Positive	371.1	668.0	15.84	361.21	650.18	15	97.33%	94.70%
	Negative	−363.1	−653.6	−18.68	−356.54	−641.77	−15	98.19%	80.30%
CT-300	Positive	486.3	875.3	21.39	437.46	787.43	15	89.96%	70.13%
	Negative	−494.4	−889.9	−17.94	−441.76	795.17	−12	89.35%	66.89%

Remark: The “positive” designation indicates the direction of the initial horizontal load applied to the upper portion of the stake, while the “negative” designation represents the opposite direction.

**Table 5:** Physical and mechanical parameters of steel reinforcement.

Designation	Model Number	Caliber/mm	Young’s Modulus/MPa	Yield Strength/MPa	Tensile Strength/MPa	Plastic Strain
Pile hoop	—	5.0	200,000	515	550	0.020
Pile prestressing steel rods	—	12.6	200,000	1280	1420	0.076
Core-filling hoop	HPB300	8.0	210,000	300	420	0.057
bearing reinforcement	HRB335	14.0	200,000	335	455	0.060
Core-filled reinforcement	HRB400	18.0	200,000	400	540	0.070
Terminal plate welded anchorage bar	HRB400	20.0	200,000	400	540	0.070
Terminal plate and connection plates			210,000	225	370	0.070

**Table 6:** Finite element results of nodal bearing capacity and stake top displacement.

Specimen Number	Ultimate Shear/kN		Limit Moment/kN·m		Limit Displacement/mm		Ratio to the Ultimate Shear Force (Limit Moment) of Type I Nodes	
	Positive	Negative	Positive	Negative	Positive	Negative	Positive	Negative
CT-I-50	189.84	−199.57	341.71	−359.23	12	−9	1.00	1.00
CT-II-50	220.48	−224.15	396.86	−403.47	9	−9	1.16	1.12
CT-III-50	95.15	−92.27	171.27	−166.09	15	−15	0.50	0.46
CT-IV-50	168.96	−167.46	304.13	−301.43	9	−9	0.89	0.84
CT-I-100	225.86	−224.16	406.55	−403.49	15	−15	1.00	1.00
CT-II-100	278.99	−277.79	502.18	−500.02	18	−18	1.24	1.24
CT-III-100	146.81	−144.30	264.26	−259.74	24	−18	0.65	0.64
CT-IV-100	220.33	−214.62	396.59	−386.32	15	−15	0.98	0.96
CT-I-150	284.42	−279.52	511.96	−503.14	12	−12	1.00	1.00
CT-II-150	330.98	−329.51	595.76	−593.12	18	−15	1.16	1.18
CT-III-150	207.66	−194.03	373.79	−349.25	15	−18	0.73	0.69
CT-IV-150	263.31	−261.96	473.96	−471.53	15	−12	0.93	0.94
CT-I-200	364.10	−364.79	655.38	−656.62	15	−15	1.00	1.00
CT-II-200	387.02	−379.63	696.64	−683.33	15	−15	1.06	1.04
CT-III-200	268.22	−263.48	482.80	−474.26	18	−18	0.74	0.72
CT-IV-200	314.30	−319.03	565.74	−574.25	15	−12	0.86	0.87
CT-I-250	398.73	−397.90	717.71	−716.22	15	−12	1.00	1.00
CT-II-250	400.77	−398.47	721.39	−717.25	15	−12	1.01	1.00
CT-III-250	310.08	−306.25	558.14	−551.25	18	−18	0.78	0.77
CT-IV-250	317.46	−324.50	571.43	−584.10	12	−12	0.80	0.82
CT-I-300	392.58	−402.84	706.64	−725.11	12	−12	1.00	1.00
CT-II-300	397.01	−395.39	714.62	−711.70	12	−12	1.01	0.98
CT-III-300	380.12	−372.75	684.22	−670.95	15	−15	0.97	0.93
CT-IV-300	320.91	−321.70	577.64	−579.06	12	−12	0.82	0.80
CT-I-350	399.07	−405.47	718.33	−729.85	12	−12	1.00	1.00
CT-II-350	401.06	−406.42	721.91	−731.56	12	−12	1.00	1.00
CT-III-350	397.07	−402.19	714.73	−723.94	12	−12	0.99	0.99
CT-IV-350	306.92	−310.14	552.46	−558.25	12	−12	0.77	0.76
CT-I-400	401.58	−409.51	722.84	−737.12	12	−12	1.00	1.00
CT-II-400	398.08	−397.74	716.54	−715.93	12	−12	0.99	0.97
CT-III-400	396.30	−405.09	713.34	−729.16	12	−12	0.99	0.99
CT-IV-400	321.57	−323.45	578.83	−582.21	12	−12	0.80	0.79
CT-I-500	400.01	−396.85	720.02	−714.33	12	−12	1.00	1.00
CT-II-500	397.99	−399.98	716.38	−719.96	12	−12	0.99	1.01
CT-III-500	401.30	−402.07	722.34	−723.73	12	−12	1.00	1.01
CT-IV-500	325.58	−321.72	586.04	−579.10	12	−12	0.81	0.81

**Table 7:** Bearing capacity of different vertical tension nodes.

Designation and Vertical Tension		Ultimate Shear/kN		Limit Moment/kN·m		Limit Displacement/mm		Ratio to the Ultimate Shear Force	
		Positive	Negative	Positive	Negative	Positive	Negative	Positive	Negative
CT-II-250	0	422.03	−432.16	759.65	−777.89	15	−12	1.00	1.00
	340	400.77	−398.47	721.39	−717.25	15	−12	0.95	0.92
	680	373.66	−369.50	672.59	−665.10	15	−12	0.89	0.86
	1020	337.59	−345.26	607.66	−621.47	15	−15	0.80	0.80
	1360	309.71	−306.96	557.48	−552.53	15	−15	0.73	0.71

## Data Availability

Restrictions apply to the availability of these data. Data were obtained from the Technology Project of the State Grid Corporation of China and are available from the authors with the permission of a third party.
